# Red blood cell lifespan in long-term hemodialysis patients treated with roxadustat or recombinant human erythropoietin

**DOI:** 10.1080/0886022X.2021.1988968

**Published:** 2021-10-17

**Authors:** Xiaowei Yang, Bing Zhao, Jing Wang, Lei Wang, Min Tao, Jing Lu, Jiangong Lin, Jing Sun, Rong Wang

**Affiliations:** a Department of Nephrology, Provincial Hospital affiliated to Shandong First Medical University, Jinan, PR China; b Department of Nephrology, Caoxian People’s Hospital, Heze, PR China

**Keywords:** Renal anemia, hemodialysis, red blood cell lifespan, Levitt’s CO breath test, roxadustat

## Abstract

**Introduction:**

A significant decrease in red blood cell (RBC) survival has been observed in patients with renal failure, which is supposed to contribute to renal anemia. The aim of this observational study was to determine RBC survival in hemodialysis (HD) patients treated with roxadustat or recombinant human erythropoietin (rhuEPO) compared with healthy persons.

**Methods:**

RBC lifespan was measured by Levitt’s CO breath test with newly developed automatic instrument ELS Tester.

**Results:**

A total of 102 patients receiving long-term HD from two independent dialysis centers enrolled in the study, of whom 62 were treated with rhuEPO and 40 were on roxadustat therapy. A total of 25 healthy participants were recruited to match HD participants according to age and sex. Median RBC survival times in rhuEPO, roxadustat, and control groups were 65.0 (25th–75th percentile, 49.5–77.3), 75.5 (25th–75th percentile, 57.3–99.3), and 108.0 (25th–75th percentile, 89.0–141.5) d, respectively. Patients treated with roxadustat had significantly longer RBC survival time than patients treated with rhuEPO (*p* < .05). In multivariate analysis of factors affecting RBC lifespan in the whole HD patients, anemia treatment drugs (rhuEPO/roxadustat) and levels of hemoglobin were the significantly independent factors. RBC survival was not found to correlate with either weekly rhuEPO dosage (*r* = –0.087, *p* = .500) or weekly roxadustat dosage (*r* = −0.267, *p* = .110) in our cohort.

**Conclusions:**

HD patients treated with roxadustat had significantly longer RBC survival time than patients treated with rhuEPO, large prospective studies with long-term follow-up are warranted to verify the results in future. **Abbreviations** RBC: red blood cell; HD: hemodialysis; rhu EPO: recombinant human erythropoietin; ESRD: end-stage renal disease; EPO: erythropoietin; ROS: reactive oxygen species; CKD: chronic kideny disease; ESAs: erythropoiesis-stimulating agents; HIF-PHD: hypoxia-inducible factor prolyl hydroxylase; CO: carbon monoxide; Hb: hemoglobin

## Introduction

Renal anemia is one of the most frequently observed complications in patients with end-stage renal disease (ESRD), and contributes substantially to patients’ morbidity and mortality [1]. Anemia associated with kidney disease is considered to result in large part from insufficient production of renal erythropoietin (EPO) and subsequent decrease of erythropoiesis. In ESRD, erythropoiesis may further be compromised by certain deficiency states, such as iron, folate, or vitamin B12 deficiency [2,3]. In addition, numerous studies have reported a significant decrease in red blood cell (RBC) survival in patients with renal failure [4–7], indicating renal anemia could at least in part result from shortened RBC survival.

The decrease of the RBC life span in renal anemia may be due to accelerated eryptosis, the apoptosis-like suicidal cell death of erythrocytes, which enables the disposal of defective cells without breaking down the cell membrane integrity and release of cytosolic material [8,9]. Prior experiments revealed that the uremic toxins vanadate [10], acrolein [11], indoxyl sulfate [12], and methylglyoxal [10] stimulate eryptosis, and the toxic uremic milieu was hypothesized to be one cause for the reduced RBC lifespan. However, in some clinical investigations, RBC lifespan was not improved along with the advancements made in chronic renal replacement therapy in spite of improved urea clearance [13]. The percentage of eryptotic cells was significantly higher after rather than prior to hemodialysis (HD), probably due to the shearing effect of the blood pump and interaction with foreign surfaces [9]. Some researchers have reported that RBC survival was similar in patients with HD and peritoneal dialysis [4]. These findings indicated that the pathogenesis of reduced RBC life span in ESRD may be complex, there may be other factors interfering with RBC survival besides the effect of plasma components and mechanical damage.

Oxidative stress, which is a permanent challenge for erythrocytes [14], is an important trigger of eryptosis [15]. Erythrocytes have powerful antioxidants to scavenge reactive oxygen species (ROS), including glutathione, superoxide dismutase, and catalase. Erythrocytes from HD patients showed increased accumulation of ROS and reduced glutathione levels which may contribute to enhanced eryptosis [16]. Iron deficiency is common in chronic kidney disease (CKD), and previous studies demonstrated that iron deficiency enhanced activity of the Ca^2+^-permeable cation channel on erythrocyte and triggered eryptosis [17]. EPO, which could bind to erythrocytes and inhibit volume-sensitive erythrocyte cation channels to prevent the breakdown of phosphatidylserine asymmetry after activation of these channels [18], is important to prolong RBC lifespan. EPO deficiency in CKD might be a contributor for the shortened RBC survival. To sum up, altered properties of erythrocytes in ESRD may participate in the triggering of eryptosis and result in the reduced RBC lifespan.

There has been little research conducted to investigate the treatments for reduced RBC survival in CKD. Observations of the effect of erythropoiesis-stimulating agents (ESAs) on RBC lifespan are controversial [19–21]. Roxadustat is a novel oral agent that is a new potent hypoxia-inducible factor prolyl hydroxylase (HIF-PHD) inhibitor developed by FibroGen. It has been approved for treating anemia in CKD patients in China and Japan [22]. It promotes the production of endogenous EPO and corrects iron metabolism disorders, and is effective in the treatment of renal anemia avoiding plasma EPO concentration peaks compared to ESAs treatment [23]. Furthermore, recent studies showed that roxadustat could perturb membrane ionic currents [24] and target genes antagonizing apoptosis and oxidative stress [25]. Roxadustat might have the potential to improve RBC longevity.

After the approval of roxadustat for marketing in the treatment of renal anemia in December 2018 in China, part of patients with maintainence HD changed anti-anemia drugs from rhuEPO to roxadustat of their own accord. The aim of this observational study was to determine RBC survival in HD patients treated with roxadustat or ESAs compared with healthy persons.

## Methods

### Study design

This was a cross-sectional observational study assessing RBC survival in HD patients treated with recombinant human erythropoietin (rhuEPO) or roxadustat and healthy participants in China. Patients were recruited independently from the Provincial Hospital Affiliated to Shandong First Medical University Dialysis Unit and Caoxian People’s Hospital Dialysis Unit. Dialysis patients were stratified according to anti-anemia drugs (rhuEPO or roxadustat) and healthy participants were included in the third arm of the study. This study was approved by the Ethics Committees of the Provincial Hospital Affiliated to Shandong First Medical University and Caoxian People’s Hospital (the approval numbers are SZRJJ:No.2021-145 and No. CH2021-003, respectively), and all patients gave written informed consent to participate. This study was conducted in accordance with the principles of the Declaration of Helsinki.

### Patients

Persons older than 18 years who had been established on maintenance HD therapy for at least 3 months were eligible to participate in this study. Eligible patients had a cause of anemia that was secondary to CKD and a blood hemoglobin concentration above 90 g/L. All participants were either using rhuEPO or roxadustat for at least 3 months. Healthy volunteers with kidney function (defined as glomerular filtration rate 60 mL/min/1.73 m^2^) and hemoglobin concentrations within the reference range were invited to enroll in this study and were matched according to age and sex to HD patients.

Exclusion criteria for all persons were active bleeding, blood transfusion of whole blood or RBCs within 30 d before study entry, concurrent malignancy, history of autoimmune diseases, and clinical evidence of hemolytic anemia or acute infective/inflammatory response at the time of study enrollment. For dialysis patients, lack of rhuEPO or roxadustat or dosage modification 30 d preceding study entry resulted in exclusion. Smokers and patients with chronic lung disease were excluded from the study.

All patients underwent dialysis two or three times a week using a Fresenius 4008B or a Fresenius 4008S machine (Fresenius Medical Care AG, Bad Homburg, Germany) and a high-flux polysulfone membrane (FX60; Fresenius Medical Care AG). Few patients received the treatment of hemodiafiltration (HDF) using a high-flux polysulfone membrane dialyzer (TS-1.6SL; Toray Medical, Tokyo, Japan) usually with one time per month. HD access was through native arteriovenous fistulas, constructed from synthetic grafts or tunneled catheters. Blood flow was set at 200–300 mL/min, and dialysate flow was set at 500 mL/min. The ultrafiltration rate was determined by each patient’s estimated dry bodyweight. All the patients in the rhuEPO group received rhuEPO intravenously, and were administered 1 or 2 times per week.

### Data collection

Clinical data, including age, gender, HD vintage, single-pool Kt/V, causes of ESRD, comorbidities, weekly EPO or roxadustat dose, post-dialysis dry weight, and 24-h urine volume were collected.

Whole blood samples were drawn at the arterial site of the AV access or tunneled catheters before HD was started on the day of RBC lifespan measurement. A complete blood count was assessed by an automatic blood cell analyzer (Sysmex, Kobe, Japan). The concentrations of sera albumin (ALB), creatinine, blood urea nitrogen (BUN), uric acid (UA), calcium (Ca), and phosphate (P) were quantified using automatic biochemical analyzer (Beckman Coulter, Brea, CA). The concentrations of sera ferritin, intact-parathyroid hormone (i-PTH), EPO, vitamin B12, and folate were quantified using automatic chemiluminescence analyzer (Beckman Coulter, Brea, CA). The doses of rhuEPO are expressed as IU/kg body weight per week. We used the EPO resistance index (ERI) as the metric of EPO resistance, defined as the weekly weight-adjusted rhuEPO dose (U/kg/week) divided by Hb level (g/dL).

### RBC lifespan measurement

Breakdown of heme results in the production of carbon monoxide (CO), and the alveolar CO concentration can be used to estimate RBC lifespan [26]. Alveolar air samples were collected pre-dialysis in the morning of the dialysis day without a fasting requirement. Briefly, after a deep inspiration, each subject held his or her breath for 10s, and then exhaled into the collection system through a mouthpiece. The collection system discarded the first 300 mL of volume, which was considered to contain dead space gas, and then directed subsequent alveolar air automatically into a foil collection bag. If needed, the procedure was repeated until the collected air sample reached the collection bag’s 1000-mL capacity. The filled bag was detached and sealed immediately. Atmospheric samples were collected just after breath sampling. Alveolar air and atmospheric samples were stored at room temperature and analyzed within 5 d. The instrument used to determine RBC lifespan was the ELS Tester (Seekya Biotec Co., Ltd., Shenzhen, China), which measures endogenous CO by non-dispersive infrared comparison of the CO content within an alveolar air sample *vs.* that in the accompanying atmospheric air sample using Levitt’s formula, the instruments CO detection limit is 150 ppb, with an accuracy of 50 ppb and precision within 50 ppb [5,27]. In a recent study, Lei Ye et al. demonstrated that the RBC lifespan values determined from the ^15^N-glycine labeling technique and the CO breath test using this instrument were significantly and strongly correlated (*r* = 0.98, *p* < .05), which suggested that the CO breath test is a reliable method for quickly determining human RBC lifespans in clinical applications [28]. Operation of this instrument involved a simple 3-step protocol: (1) addition of alveolar and environmental samples; (2) inputting of (Hb) information; and (3) pressing a start measurement button. The measurements and calculations for each assessment were completed within 15 min. Women patients should avoid the menstrual period when monitoring RBC lifespan.

### Sample size calculation

The sample size was determined based on our preliminary study of RBC survival on 15 patients in each group. In that study, mean RBC survival in those with rhuEPO was 70.3 (± standard deviation, SD 26.8 d) compared to 86.5 ± 20.0 d in those with roxadustat. The sample size required to obtain 85% power at *α* error level of 5% was calculated to be at least 40 subjects per group.

### Statistical analysis

Statistical software SPSS version 25.0 (SPSS, Chicago, IL) was employed for statistical analysis. Quantitative data were expressed as mean ± SD, median and 25th–75th percentile, median and range, or number (%). For comparison of clinical features and RBC lifespan of patients, t-tests, Mann–Whitney U test, Kruskal–Wallis test, *χ*^2^ test, and one-way ANOVA were used with Dunnett’s test for multiple for multiple comparisons. The linear regression model was applied to assess the factors influencing the RBC survival in HD patients. Only variables with *p* < .05 in the univariate linear regression analysis were used in the multiple linear regression. Categorial variables, including sex, anti-anemia drugs, and diabetes, were transformed into dichotomous dummy variables (male:0, female:1; rhuEPO:0, roxadustat:1; no diabetes:0, diabetes:1), skewed continuous variables were logarithmically transformed into normal distributions. Statistical significance was considered as *p* < .05.

## Results

### General characteristics of the study participants

A total of 102 patients receiving long-term HD from two independent dialysis centers participated in this observational study, of whom 62 were treated with rhuEPO and 40 were on roxadustat therapy. A total of 25 healthy participants (15 men and 10 women) were recruited to match HD participants according to age and sex. Mean age of the healthy participants was 52.7 ± 12.4 years with serum creatinine levels at 63.9 ± 15.7 μmol/L. Hemoglobin concentrations in healthy controls were significantly higher than for all HD patients (142.6 ± 11.0 *vs*. 113.6 ± 13.6 g/L; *p* < .001).

General characteristics for HD participants are listed in Table 1. At the time of RBC lifespan measurement, there were no statistical differences in the distributions of age, sex, HD vintage, weekly HD hours, single-pool Kt/V, and causes of ESRD between rhuEPO and roxadustat groups. Hemoglobin concentrations were not significantly different between patients treated with rhuEPO and roxadustat (112.0 ± 12.9 *vs*. 116.1 ± 14.5 g/L; *p* = 0.139), and the serum levels of creatinine, ALT, Ca, P, bicarbonate, PTH, EPO, vitamin B12, folate, and CRP were also comparable between the two groups. In this cohort, patients received roxadustat had significantly higher levels of BUN and lower levels of β2-MG compared with patients treated with rhuEPO. In our cohorts, no patient received oral iron tablets, and in the roxadustat group patients received fewer doses of intravenous iron supplementation. Then ferritin levels were significantly lower than that of patients in rhuEPO group (168.8 ± 138.3 *vs*. 318.3 ± 170.3 ng/ml, *p* < .0001).

**Table 1.** General characteristics of the study HD participants.

Variable	rhuEPO	Roxadustat	
	At the RBC lifespan measurement *n* = 62	Before the use of roxadustat *n* = 35	At the RBC lifespan measurement *n* = 40
Age (mean ± SD) (years)	57.0 ± 13.4	52.4 ± 13.0	53.9 ± 13.3
Sex (male/female)	38/24	23/17	23/17
Cause of ESRD			
Diabetes (%)	18 (29.0)	13 (32.5)	13 (32.5)
Hypertension (%)	4 (6.5)	1 (2.5)	1 (2.5)
Glomerulonephritis (%)	26 (41.9)	18 (45.0)	18 (45.0)
Polycystic disease (%)	2 (3.2)	0 (0)	0 (0)
Other (HSPN, unknown) (%)	12 (19.6)	8 (20%)	8 (20%)
HD vintage (mean ± SD) (months)	51.9 ± 41.2	41.1 ± 35.6	42.6 ± 37.2
Weekly HD hours (mean ± SD)	11.7 ± 1.0	11.5 ± 1.3	11.5 ± 1.3
Single-pool Kt/V	1.25 ± 0.17	1.22 ± 0.23	1.23 ± 0.21
Residual urine volume (median, range) (ml)	100 (0, 1200)	100 (0, 1000)	100 (0, 1000)
Weekly rhuEPO dose (mean ± SD) (U)	10,004 ± 3749	9500 ± 3794.3	
Weekly roxadustat dose (mean ± SD) (mg)			297 ± 123
Roxadustat duration time (median, range) (month)			6 (3 ∼ 12)
Intravenous iron supplementation (%)	40 (64.5)	24 (68.6%)	15 (37.5)**
Hemoglobin (mean ± SD) (g/L)	112.0 ± 12.9	107.3 ± 29.6	116.1 ± 14.5
Reticulocyte ratio (mean ± SD) (%)	1.94 ± 0.81		1.90 ± 0.93
WBC (mean ± SD) (10^9^/L)	5.95 ± 1.83	6.78 ± 1.87	6.19 ± 1.90
PLT (mean ± SD) (10^9^/L)	177.8 ± 45.5	176.9 ± 58.4	187.7 ± 58.1
SCR (mean ± SD) (μmol/L)	875.8 ± 264.7	824.1 ± 148.6	846.5 ± 205.9
BUN (mean ± SD) (μmol/L)	25.8 ± 7.6	25.1 ± 4.2	29.1 ± 6.3
β2-MG (mean ± SD) (mg/L)	27.9 ± 4.1	23.7 ± 3.0***	23.7 ± 7.8**
ALB (mean ± SD) (g/L)	41.2 ± 4.4	39.7 ± 3.1	40.7 ± 4.8
Ca (mean ± SD) (mmol/L)	2.27 ± 0.22	2.33 ± 0.18	2.23 ± 0.21
P (mean ± SD) (mmol/L)	1.89 ± 0.56	1.99 ± 0.38	2.00 ± 0.51
Bicarbonate (mean ± SD) (mmol/L)	20.4 ± 4.0	21.6 ± 1.71	19.4 ± 2.8
Ferritin (mean ± SD) (μg/L)	318.3 ± 170.3	341.3 ± 186.8	168.8 ± 138.3***
Serum iron (mean ± SD) (μmol/L)	14.4 ± 4.8	14.2 ± 3.7	14.5 ± 4.6
TIBC (mean ± SD) (μmol/L)	44.0 ± 5.6	47.8 ± 6.7	53.2 ± 9.5***
Transferrin saturation (mean ± SD) (%)	33.3 ± 11.7	29.6 ± 10.0	27.6 ± 8.4
PTH (mean ± SD) (pg/mL)	335.1 ± 217.4	349.9 ± 348.5	280.2 ± 280.6
CRP (mean ± SD) (mg/L)	6.64 ± 17.1	1.85 ± 1.37	3.02 ± 4.12
Serum EPO levels (mean ± SD) (mIU/mL)	20.22 ± 33.20		19.19 ± 37.33
Serum folate levels (mean ± SD) (ng/mL)	7.94 ± 4.11		8.38 ± 4.65
Serum vitamin B12 levels (mean ± SD) (pg/mL)	589.61 ± 447.38		659.67 ± 534.31

SD: standard deviation; HSPN: Henoch–Schonlein syndrome; HD: hemodialysis; WBC: white blood cells; PLT: platelets; SCR: serum creatinine; BUN: blood urea nitrogen; β2-MG: β2 microglobulin; ALB: albumin; Ca: calcium; P: phosphate; TIBC: total iron-binding capacity; PTH: parathyroid hormone; CRP: C-reactive protein; EPO: erythropoietin

Data are provided as median (range) or mean (SD).

**p < .01 and ***p < .001, compared with the rhuEPO group with Dunnett’s test for multiple comparison correction.

Although the patients changed their anti-anemia drugs from rhuEPO to roxadustat totally voluntarily in our dialysis units, we also reviewed the baseline data of our roxadustat group patients before the use of roxadustat. Five patients of the roxadustat group received roxadustat at the beginning of HD therapy, so 35 patients had the baseline data. The weekly rhuEPO dosages, the hemoglobin concentrations, the serum ferritin levels, and intravenous iron supplementation dosages of these patients were comparable with the rhuEPO group. The serum β2-MG levels were also significantly lower of the roxadustat patients before the use of roxadustat compared with that of rhuEPO group (Table 1).

### RBC lifespan of the study participants

**Figure 1.**
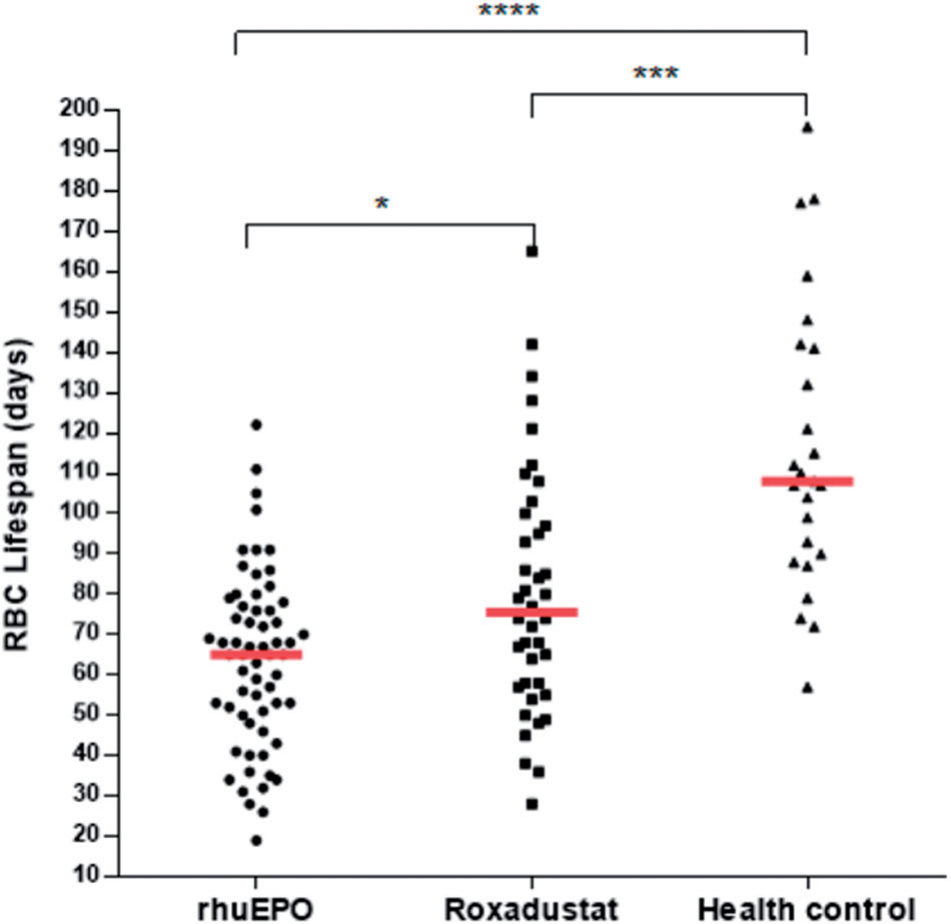
Scatter plot shows red blood cell (RBC) survival in days in hemodialysis (HD) patients treated with rhuEPO or roxadustat, and controls. Group median values are represented by a full red horizontal line. For comparison RBC lifespan among the three groups, Kruskal–Wallis test was used with Bonferroni correction for multiple comparisons. *p < .05, ***p < .001, and ^****^ *p* < .0001.

Figure 1 shows RBC survival of all participants stratified into HD patients treated with rhuEPO or roxadustat, and healthy controls. In this study, median RBC survival times in rhuEPO, roxadustat, and control groups were 65.0 (25th–75th percentile, 49.5–77.3), 75.5 (25th–75th percentile, 57.3–99.3), and 108.0 (25th–75th percentile, 89.0–141.5) d, respectively. There was a significant difference in RBC survival in HD patients compared with participants with kidney function in the reference range (*p* < .001). Patients treated with roxadustat had significantly longer RBC survival time than patients treated with rhuEPO (*p* < .05). The median RBC lifespan of the roxadustat group was 16.2% higher than that of the rhuEPO group,

### Factors associated with RBC lifespan in HD patients

**Figure 2.**
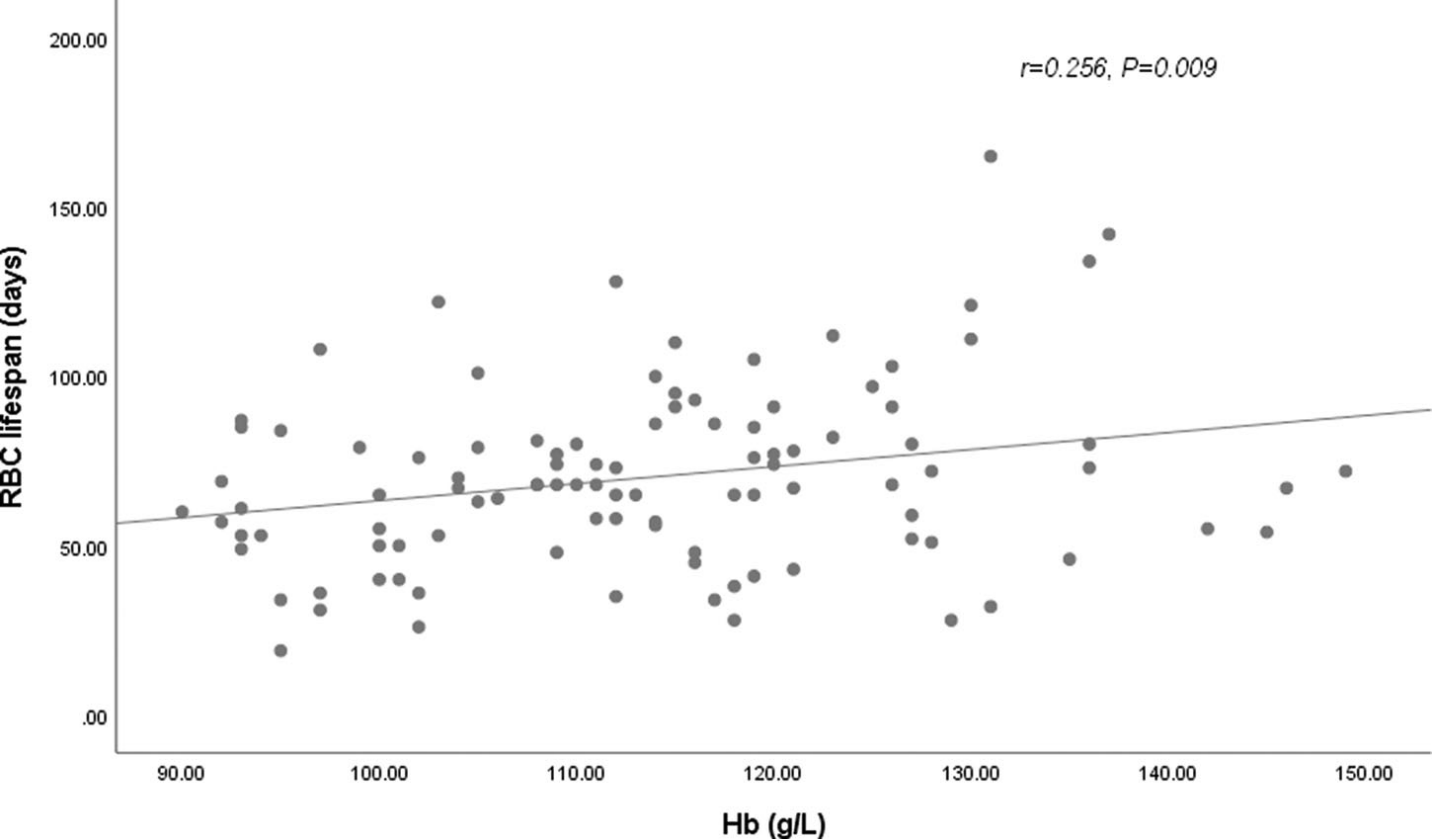
Relationship between RBC lifespan and hemoglobin (Hb). A significant positive correlation was observed between the RBC lifespan and Hb levels in 102 hemodialysis patients in a univariate linear correlation analysis (*r* = 0.256, *p* = .009).

**Figure 3.**
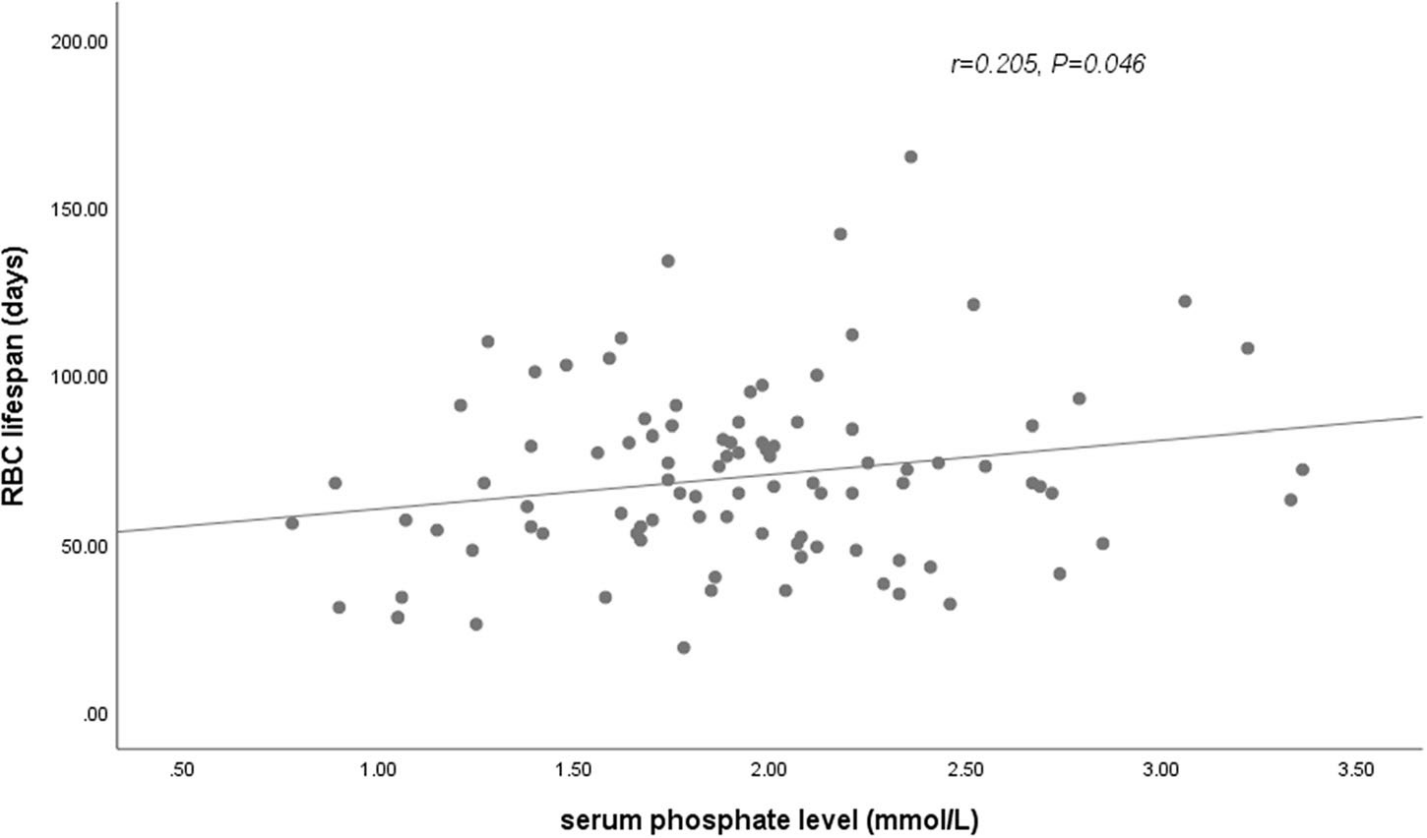
Relationship between RBC lifespan and serum phosphate levels. A significant positive correlation was observed between the RBC lifespan and phosphate levels in 102 hemodialysis patients in a univariate linear correlation analysis (*r* = 0.205, *p* = .046).

In our cohort, longer RBC survival was observed in HD patients treated with roxadustat compared with patients on rhuEPO therapy. Since previous studies have reported that RBC lifespan were correlated with other clinical and laboratory parameters, such as levels of BUN, SCR, and ALB, univariate and multiple linear regression analysis were further conducted to determine the factors influencing the RBC lifespan of HD patients in our cohort (Table 2 and Table 3). In univariate analysis, anemia treatment drug (rhuEPO or roxadustat), levels of hemoglobin (Figure 2), and phosphate (Figure 3) were associated with the RBC survival time with statistical significance (*p* < .05). The RBC lifespan was not associated with sex, age, HD vintage, exogenous iron dosage, serum levels of BUN, SCR, β2-MG, Ca, ferritin, or transferrin saturation. In the further multivariate analysis, anemia treatment drugs (rhuEPO or roxadustat), and levels of hemoglobin were the significantly independent factors associated with RBC lifespan in HD patients.

**Table 2.** Univariate linear regression analysis of factors affecting the RBC lifespan.

	Non-standardized coefficients		Standardized coefficient Beta	*t*	*p*
	*B*	Standard error			
Sex					
Male	0 (reference)				
Female	9.395	5.334	0.173	1.761	.081
Age (year)	−0.189	0.199	−0.095	−0.953	.343
HD vintage (month)	−0.093	0.066	−0.139	−1.399	.165
Weekly HD hours (hour)	−1.807	2.344	−0.077	−0.774	.440
Hemoglobin (g/L)	0.503	0.190	0.256	2.652	.009
Reticulocyte ratio (%)	−3.617	3.192	−0.125	−1.133	.260
SCR (μmol/L)	0.005	0.011	0.041	0.399	.691
BUN (mmol/L)	0.555	0.377	0.151	1.475	.143
β2-MG (mg/L)	−0.394	0.486	−0.089	−0.810	.421
ALB (g/L)	−0.548	0.617	−0.093	−0.888	.377
Ca (mmol/L)	16.380	12.672	0.133	1.293	.199
P (mmol/L)	10.232	5.053	0.205	2.025	.046
Bicarbonate (mmol/L)	−0.593	0.789	−0.079	−0.752	.454
Ferritin (μg/L)	0.006	0.016	0.040	0.373	.710
Transferrin saturation (%)	0.022	0.382	0.008	0.057	.955
PTH (pg/mL)	0.001	0.011	0.010	0.097	.923
Iron supplement dose (mg/month)	−0.025	0.027	−0.091	−0.918	.361
CRP (mg/L)	−0.341	0.279	−0.166	−1.224	.226
Residual urine volume (mL)	0.005	0.010	0.045	0.442	.659
Anti-anemia drug					
rhuEPO	0 (reference)				
Roxadustat	16.474	5.184	0.303	3.178	.002
Diabetes					
Without diabetes	0 (reference)				
With Diabetes	−1.683	5.721	−0.029	−0.294	.769

HD: hemodialysis; SCR: serum creatinine; BUN: blood urea nitrogen; β2-MG: β2 microglobulin; ALB: albumin; Ca: calcium; P: phosphate; PTH: parathyroid hormone; CRP: C-reactive protein

**Table 3.** Multiple linear regression analysis of factors affecting the RBC lifespan.

	Non-standardized coefficients		Standardized coefficient Beta	*t*	*p*
	*B*	Standard error			
Constant	2.593	23.348		0.111	.942
Anti-anemia drug					
rhuEPO	0 (reference)				–
roxadustat	12.977	5.321	0.239	2.439	.017
Hemoglobin (g/L)	0.409	0.197	0.204	2.080	.040
Phosphate (mmol/L)	8.019	4.839	0.161	1.657	.101

### Correlations of RBC lifespan and rhuEPO/roxadustat dosage

In our 62 HD patients treated with rhuEPO, RBC survival did not correlate with weekly rhuEPO dosage (*r* = −0.087, *p* = .500) or rhuEPO resistance index (weekly rhuEPO dose adjusted by weight and hemoglobulin level) (*r* = −0.166, *p* = .196). In our 40 HD patients treated with roxadustat, RBC survival did not correlate with weekly roxadustat dosage (*r* = −0.267, *p* = .110).

## Discussion

This is the first study to compare RBC lifespan in maintenance HD patients treated with rhuEPO and roxadustat. We enrolled HD patients from two independent dialysis centers, and confirmed that RBC survival was decreased significantly in HD patients compared with those with kidney function in the reference range. Interestingly, HD patients treated with roxadustat had significantly longer RBC longevity compared with patients on rhuEPO therapy, although still much lower than that of the healthy control.

In this observational study, patients enrolled in the roxadustat group were comparable in the distribution of age, sex, HD vintage, weekly HD hours, single-pool Kt/V, causes of ESRD and levels of hemoglobin, creatinine, ALB, Ca, P, bicarbonate, PTH, and CRP to patients enrolled in the rhuEPO group. Patients in roxadustat group had significantly higher levels of BUN and lower levels of β2-MG. Patients with roxadustat received less exogenous iron supplementation, and had lower levels of serum ferritin. Previous studies have reported that HD dose indices, such as BUN and SCR, were positively correlated with the RBC lifespan [6,7], and abnormal iron metabolism could result in accelerated eryptosis [17]. Whether the difference of RBC lifespan in rhuEPO and roxadustat groups caused by anti-anemia drugs or by differences in BUN and iron metabolism remains a question. Then, we further conducted linear regression analysis to assess the factors influence RBC lifespan in all the HD patients.

We found that exogenous iron dosage, serum levels of BUN, SCR, β2-MG, Ca, ferritin, or transferrin saturation were not associated the RBC lifespan in our cohort. Anemia treatment drug (rhuEPO/roxadustat) was the significant factor associated with RBC lifespan either in the univariate or multivariate analysis. These results suggest that roxadustat seemed to be more effective in improving RBC longevity than rhuEPO. In our study, the median RBC lifespan of roxadustat group was 16.2% (10.5 d) higher than that of rhuEPO group. Since roxadustat was a newly approved drug to treat renal anemia in China, the median duration time of roxadustat treatment in our patients was only 6 months. Polenakovic and Sikole suggested that the effect of rhuEPO on RBC survival might be related to the duration of treatment [19]. Whether RBC survival getting longer accompanied with the extension of roxadustat treatment duration needs further long-term follow-up study.

A variety of factors have been reported to associate with RBC lifespan with inconsistent results. While not being part of the primary data analysis, we found a weak positive correlation between serum phosphate levels and RBC lifespan, which was not confirmed in multivariate analysis. In animal experiment, phosphate depletion contributes to the enhanced vulnerability and accelerated sequestration of erythrocytes [29]. The correlation of RBC lifespan with phosphate in patients with CKD warrants further studies.

Although reduced RBC lifespan was considered to be a contributor to renal anemia, observations of the correlation between EPO responsiveness and RBC survival are controversial [4,7]. In our cohort, fewer use of roxadustat or rhuEPO was also not observed in patients with longer RBC lifespan. There might be two explanations for this. First, healthy controls exhibit a wide range of RBC lifespan, the balance of hematopoiesis and eryptosis might be complex. The correlation of the falling value rather than the absolute value of the RBC lifespan to anti-anemia drug responsiveness in HD patients might be worth further study. Second, there were plenty of factors interfering with drug responsiveness, such as secondary hyperparathyroidism and dialysis adequacy. The correlation between the dosage of anti-anemia drugs and RBC survival should be evaluated in large sample-sized and well-controlled clinical studies.

The major limitation of our study is that it was a cross-sectional observational study with small sample size, a cause-effect relationship could not be established. Large prospective studies with long-term follow-up are needed to verify the results in future.

## Conclusion

In summary, this preliminary cross-sectional observational study showed that HD patients treated with roxadustat had significantly longer RBC survival time than patients treated with rhuEPO.
